# Right Atrial Thrombosis Provoked by Central Venous Catheter: A Case Report

**DOI:** 10.7759/cureus.9027

**Published:** 2020-07-06

**Authors:** Kalyan Prudhvi, Kris Kumar, Jayasree Jonnadula, Rajesh Janardhanan

**Affiliations:** 1 Internal Medicine/Nephrology, Albert Einstein College of Medicine - Montefiore Medical Center, New York, USA; 2 Cardiology, Oregon Health and Science University School of Medicine, Portland, USA; 3 Internal Medicine, Banner University Medical Center, Tucson, USA; 4 Cardiology, Banner University Medical Center, Tucson, USA

**Keywords:** intracardiac thrombi, atrial thrombosis, ventricular thrombosis

## Abstract

Intracardiac thrombi are not uncommon, but right atrial (RA) thrombi are exceedingly rare. Thrombi can lead to a variety of complications, such as systemic and pulmonary embolism. While various imaging modalities are helpful in the diagnosis, an echocardiogram is the most commonly used one. Principle management of the condition involves anticoagulation. However, management can vary among different patient groups, depending on the location and size of thrombi. We present a case of an RA thrombosis due to dilation of the atria and trauma from an infected central venous catheter in a patient with a past medical history of pulmonary artery hypertension (PAH).

## Introduction

Intracardiac thrombi may lead to systemic complications depending on their location, size, and associated conditions [1]. Clinical presentation may vary from being asymptomatic to systemic and pulmonary embolism, stroke, and sudden cardiac death. Echocardiography is the most commonly used diagnostic modality followed by cardiac magnetic resonance imaging (CMR) and cardiac CT. Treatment options include anticoagulation and surgical thrombectomy in select patients. We present a case of right atrial (RA) thrombi associated with an infected central venous catheter, which was managed with anticoagulation and short-term antibiotics with good resolution.

## Case presentation

A 32-year-old female with idiopathic pulmonary arterial hypertension (PAH) on treprostinil infusion presented to the emergency room with complaints of pain, redness, and purulent discharge from the infusion catheter insertion site on the right-sided chest wall. She had undergone right subclavian Hickman catheter placement three months prior for the central venous infusion of treprostinil as she had been intolerant to the subcutaneous route. She had been treated with oral trimethoprim-sulfamethoxazole for four days as an outpatient prior to her presentation with no improvement in her symptoms.

**Figure 1:**
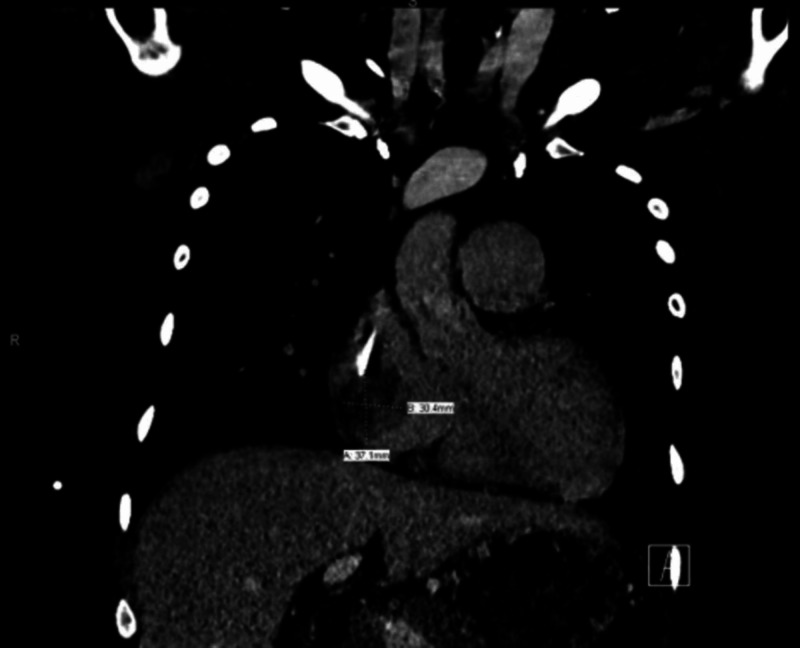
Coronal view of contrast-enhanced CT of the chest The image shows right atrial filling defect measuring 3.7 x 3.0 cm CT: computed tomography

The patient was hospitalized and started on broad-spectrum intravenous antibiotics (vancomycin and cefepime) after blood cultures were drawn. Her CT chest with contrast for evaluation of abscess around the catheter site revealed an RA filling defect (Figure 1).

**Figure 2:**
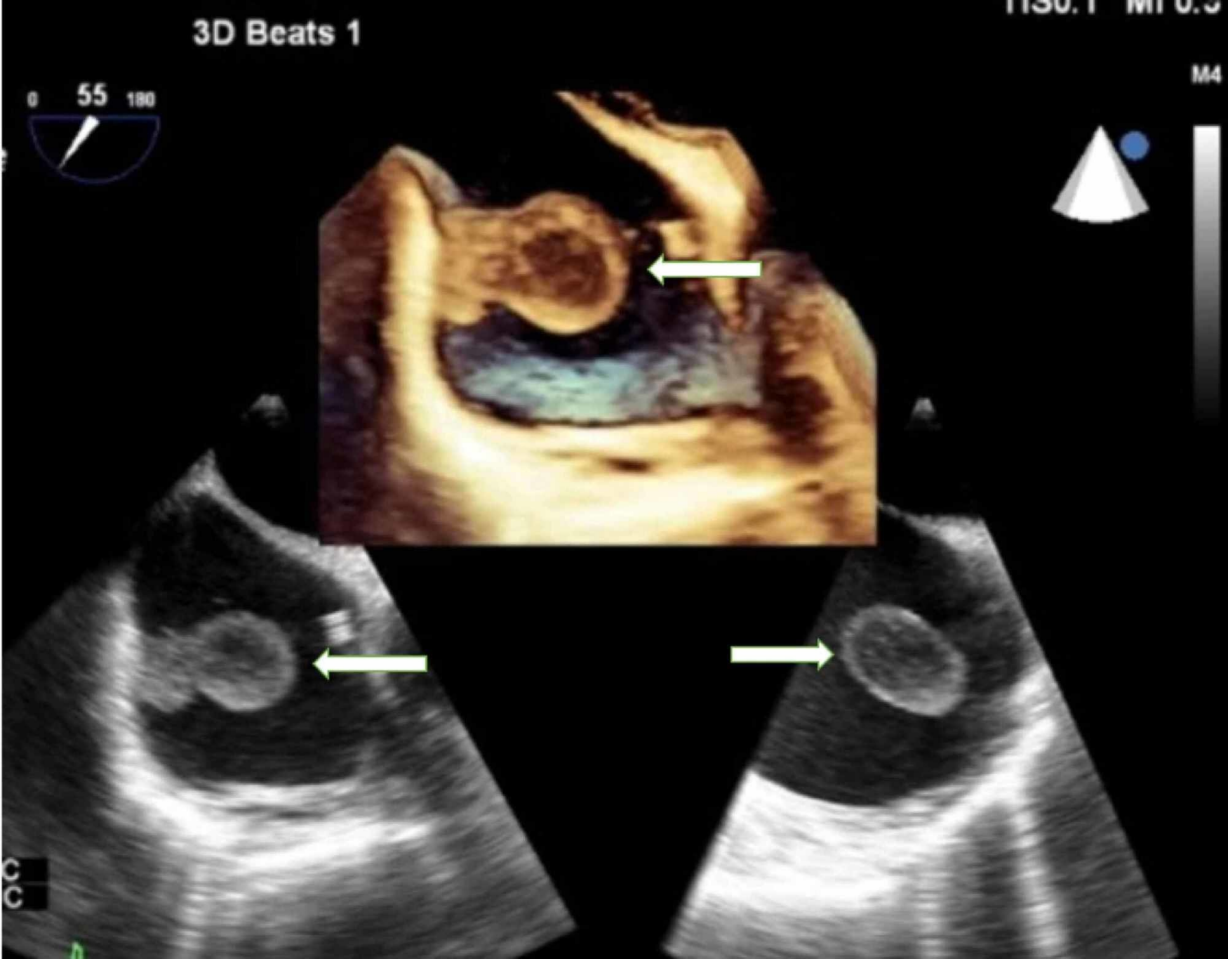
Two-dimensional transesophageal echocardiogram The image shows the mid-esophageal bicaval view of the atrium demonstrating RA thrombus arising from the right atrial free wall, measuring 1.9 x 3.6 cm (arrows). Inset shows three-dimensional TEE showing RA thrombi (arrow) TEE: transesophageal echocardiogram; RA: right atrial

**Figure 3:**
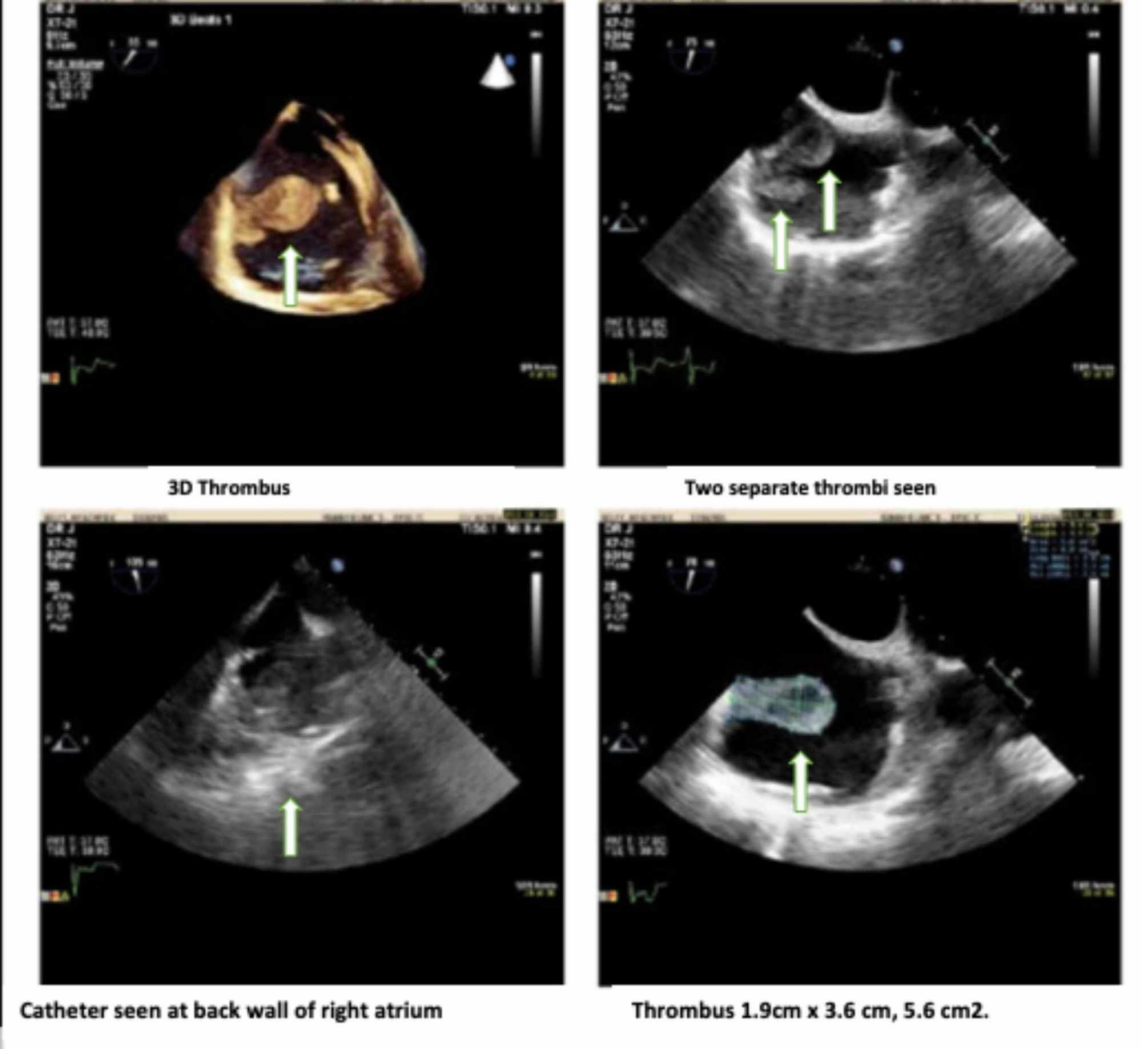
Three-dimensional and Two-dimensional views of thrombi The catheter tip is separate and unattached to the thrombi; the dimensions of the thrombus size are also displayed

Transthoracic echocardiogram (TTE) showed an echolucent structure in the right atrium. Due to her history of recurrent hemoptysis and subsequent embolization of aortopulmonary collaterals with metal coils, cardiac magnetic resonance imaging (CMR) could not be employed to further evaluate the echolucent structure. The patient underwent three-dimensional transesophageal echocardiography (3D-TEE) for further evaluation of the echolucent structure, which revealed two mobile, pedunculated thrombi, both attached to the RA free wall and opposite to the free tip of the central venous catheter. The thrombi were not attached to the catheter or the tricuspid valve (Figures 2, 3).

Thrombi formation was attributed to the constant trauma to the RA wall from the indwelling catheter in an already dilated right atrium secondary to PAH. A multidisciplinary team approach to care including cardiology, pulmonary, and hematology recommended removal of the central line and anticoagulation with warfarin, in order to maintain target international normalized ratio (INR) of 2-3 until the resolution of thrombi.

Central venous catheter was removed and the patient was transitioned to subcutaneous treprostinil infusion. The catheter tip grew oxacillin- susceptible *Staphylococcus aureus,* while two sets of blood cultures drawn 48 hours apart demonstrated no bacterial growth. Since concurrent infection of RA thrombi could not be excluded, the patient was treated with two weeks of intravenous cefazolin therapy. A follow-up TTE two weeks after completion of the antibiotic course showed resolution of the intracardiac thrombi.

## Discussion

Thrombi within the cardiac chambers may be atrial, ventricular, or device-/prosthetic valve-related. Thrombus formation is caused by the alterations in the Virchow’s triad (intracardiac chamber wall, blood flow, and blood components) (Table 1) [1]. Echocardiography, cardiac CT, and CMR are useful tools in the diagnosis of intracardiac thrombi. Although anticoagulation is vital in the management, treatment varies among different subgroups.

**Table 1:** Etiology of intracardiac thrombi classified according to Virchow's triad

Cause	Description
Chamber wall causes	Myocardial infarction (akinesis or hypokinesis), dilated left atrium (diastolic dysfunction), dilated right atrium (pulmonary arterial hypertension), ventricular aneurysms, dilated cardiomyopathy, Takotsubo cardiomyopathy, stress-related cardiomyopathy, peripartum cardiomyopathy, myocardial non-compaction, endocardial injury due to central venous catheters, pacemakers, defibrillator leads, left ventricular assist devices (LVAD), and atrial septal aneurysm
Abnormal flow states	Heart rate or rhythm disturbances (atrial fibrillation, atrial flutter, ventricular fibrillation, or ventricular tachycardia), increased turbulence due to prosthetic valves, valve stenosis (mitral, tricuspid or aortic) and mitral annular calcification
Blood component causes	Hypercoagulable states, protein C and/or S deficiency, antiphospholipid antibody syndrome, and paraganglioma due to catecholamine excess

Atrial thrombus

RA thrombi are less frequent compared to left atrial (LA) or LA appendage (LAA) thrombi related to atrial fibrillation (AF). LA thrombi carry a higher risk of systemic thromboembolism and stroke. AF increases the risk of thrombus formation in the left atrium more than in the right [1]. It has been proposed that platelet reactivity is greater in the left atrium compared to either right atrium or peripheral circulation [2]. Wider size and lack of anatomic remodeling of the RA appendage are also thought to be the reasons for RA thrombi being rare when compared to LA thrombi [3].

RA thrombus may be formed within the RA cavity itself or it may be the expulsion of peripheral venous thrombosis. The presence of RA thrombus should lead to a strong suspicion for venous thrombus extension or dislodgement. Depending on the size and extent of thrombosis, the clinical presentation can vary from being asymptomatic to massive pulmonary embolism and sudden death [4].

Diagnosis

TTE is the initial diagnostic test of choice when an intracardiac thrombus is suspected. However, TEE is better at identifying atrial thrombus, especially LA thrombi. TEE has better sensitivity (93%) and specificity (100%) for diagnosing LA thrombus when compared to TTE (sensitivity: 53%) [5]. Although CT has better sensitivity and specificity, echocardiography is still preferred as CT is limited by radiation and intravenous contrast risks. CMR provides the evaluation of pulmonary venous anatomy and can be the single best study to obtain complete information prior to pulmonary vein isolation. Among various CMR modalities, long TI-delayed enhancement CMR has the highest diagnostic accuracy (99.2%), sensitivity (100%), and specificity (99.2%) [6].

Ventricular thrombus

Left ventricular (LV) apical or mural thrombi are common within the LV cavity. LV thrombi are commonly seen after left anterior descending artery (LAD) occlusion, resulting in anterior wall myocardial infarction (MI) and anterior or apical LV aneurysms [7]. LV regional wall motion abnormalities, reduced contractility, and sluggish blood flow predispose to thrombogenesis. Incidence of LV thrombus is more common with anterior wall MI, male gender, systemic hypertension, and reduced systolic function. Heart failure with reduced ejection fraction, non-ischemic, dilated cardiomyopathy, takotsubo cardiomyopathy, and ventricular non-compaction are other notable causes for ventricular thrombi. For the diagnosis of ventricular thrombus, TTE coupled with contrast enhancement and color flow has better accuracy than TEE. Incomplete visualization of LV apex is a limitation for TTE when compared to TEE in diagnosing LV thrombus.

Device-related thrombus

Prosthetic Valve-Related Thrombus

Prosthetic valve thrombosis is more common with mechanical valves when compared with bioprosthetic valves. Right-sided valve thrombosis is more common than left-sided valve thrombosis, and mitral valve-related thrombi are more common than aortic valve thrombi. Incidence of prosthetic valve thrombosis increases further during the immediate postoperative period after valve replacement, when anticoagulation is held for postoperative bleeding concerns or if underlying hypercoagulable states such as malignancy or pregnancy are present. Diagnosis is mainly via echocardiographic evidence of thrombus, reduced effective orifice area, elevated gradients across the prosthetic valves, decreased mobility, or immobile valve leaflets. Cinefluoroscopy is a gold standard for detecting mechanical valve thrombosis; the size of the thrombus determines the risk of systemic embolism, with surgery being the standard of care for obstructing thrombus or if thrombus size is >0.8 cm^2^. TEE may help to guide when it comes to choosing between surgery and thrombolytic therapy and for patient’s follow-up. Thrombolytic therapy as a second-line therapy may be considered in patients with contraindications for surgery as it has good success rates in patients with non-obstructive thrombi, right-sided thrombi, or thrombi of relatively small size (<0.5 cm).

Lead-Associated Thrombus

Implantable device electrode leads are associated with mobile thrombi in up to 30% of patients undergoing ablation. They are better identified with intracardiac echocardiography (ICE) than TTE. Lead-associated thrombosis is more often seen in the right atrium when compared to the right ventricle (RV) [8]. Lead thrombosis may also lead to pulmonary embolism.

Left Ventricular Assist Device (LVAD)-Associated Thrombus

Incidence of LVAD thrombosis is estimated at 2-8% and is usually related to surgical implantation techniques, internal shear stress, device materials, infection, concomitant erythropoietin use, underlying hypercoagulable states, and/or inadequate anticoagulation. Initial medical management of suspected device thrombosis involves the initiation of a higher level of anticoagulation when the patient is already on anticoagulation prophylaxis via intravenous unfractionated heparin with monitoring of activated partial thromboplastin time (aPTT) with a higher goal or factor Xa assay [9]. Bivalirudin is preferred in bridging heart transplant patients, and low-molecular-weight heparin (LMWH) may be used with the transition from heparin in LVAD patients for the prevention of thrombosis. Vitamin K antagonists (VKAs) are contraindicated in this patient population.

Management

Medical Management

Unfractionated heparin, LMWH, and VKAs are the commonly used anticoagulation options; case studies have also reported the use of target-specific oral anticoagulants (TSOAC). Intravenous high-dose heparin for 7-22 days has been shown to completely resolve LV thrombi without embolic or bleeding complications though the need for prolonged hospitalization for intravenous heparin therapy is a significant limitation. In patients presenting with ST-elevation MI (STEMI) with evidence of LV thrombus, management with systemic anticoagulation in addition to antiplatelet therapy is recommended to prevent systemic embolic complications. Bleeding risk increases with triple antithrombotic therapy after a percutaneous coronary intervention (PCI), and the risk-to-benefit ratio needs to be considered on a case-by-case basis.

Initial bridging with heparin or low molecular heparin with VKAs to reach a standard INR goal of 2-3 is a commonly used anticoagulation regimen. Case reports have shown TSOACs having additional fibrinolytic activity in addition to anticoagulation effects, and they have been shown to be effective in the resolution of thrombus [10].

Surgical Removal

No definitive evidence is available comparing the efficacy of anticoagulation versus surgical resection. Surgical thrombectomy is warranted for large and mobile thrombi and recurrent thromboembolism while the patients are already on optimal anticoagulation.

## Conclusions

Early diagnosis and management of intracardiac thrombi are key to preventing systemic complications. The diagnostic accuracy of various cardiac imaging modalities varies with the location of thrombi. Although systemic anticoagulation is vital, treatment guidelines are vague regarding its duration and choice; hence, it should be considered carefully on a case-by-case basis. Our patient had RA thrombus related to the central venous catheter, which resolved after catheter removal and one month of therapeutic anticoagulation with warfarin.
